# Phenotypic insecticide resistance status of the *Culex pipiens* complex: a European perspective

**DOI:** 10.1186/s13071-022-05542-x

**Published:** 2022-11-12

**Authors:** Stien Vereecken, Adwine Vanslembrouck, Isabelle Marie Kramer, Ruth Müller

**Affiliations:** 1 The Unit of Entomology, Department of Biomedical Sciences, Institute of Tropical Medicine, 2000 Antwerp, Belgium; 2 Institute of Occupational, Social and Environmental Medicine, Goethe University, Frankfurt, Germany; 3 Senckenberg Biodiversity and Climate Research Centre, Frankfurt, Germany

**Keywords:** *Culex pipiens molestus*, Belgium, Vector control, WHO susceptibility test, *Bacillus thuringiensis israelensis*, Bioassays, Phenotypic resistance, Permethrin, Deltamethrin, DDT

## Abstract

**Background:**

The common house mosquito *Culex pipiens* is known to be a major vector for West Nile virus. In order to decrease risks of West Nile virus outbreaks in Europe, insecticides and the bio-larvicide *Bacillus thuringiensis israelensis* (Bti) are commonly used for vector control. Alarmingly, insecticide resistance has been reported in *Cx. pipiens* populations from Southern Europe and several countries neighbouring Europe. For Central and Northern Europe, however, the phenotypic insecticide resistance status of *Cx. pipiens* has not yet been investigated.

**Methods:**

A literature review was performed to assess the geographical distribution of insecticide resistance in *Cx. pipiens*. To fill the gap of knowledge for Central and Northern Europe, WHO susceptibility tests with permethrin, deltamethrin, malathion, bendiocarb and DDT and a larval toxicity test with Bti were performed with a *Cx. pipiens* population from Belgium, a country in Central Europe.

**Results:**

This research provides the first evidence of widespread phenotypic insecticide resistance in *Cx. pipiens*. In general, *Cx. pipiens* developed resistance against multiple insecticides in several countries. Another Cx. pipiens population from Belgium was tested and showed insecticide resistance against deltamethrin, permethrin, DDT and possibly against bendiocarb. The bio-larvicide Bti caused lower mortality than reported for other *Cx. pipiens* populations in the literature.

**Conclusions:**

These results indicate the urgent need for insecticide resistance monitoring against commonly used adulticides and larvicides in Europe, for the translation of knowledge gained regarding the limited efficiency and availability of insecticide into EU legislation and the need for innovative non-chemical vector control tools in order to counter the widespread insecticide resistance in *Culex* populations.

**Graphical abstract:**

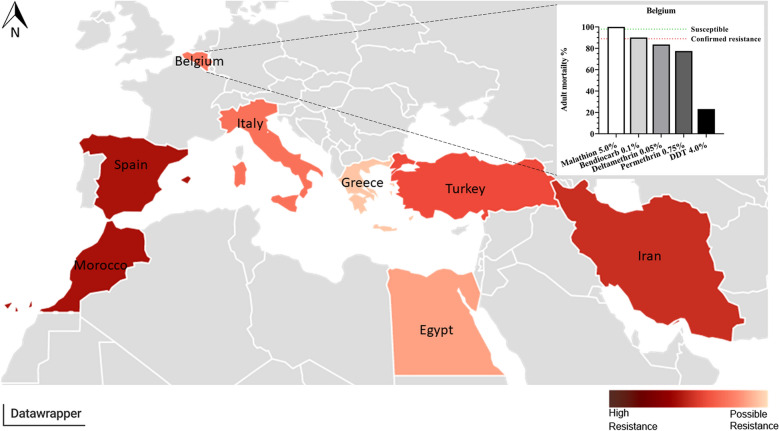

**Supplementary Information:**

The online version contains supplementary material available at 10.1186/s13071-022-05542-x.

## Background

The arboviruses West Nile virus (WNV) and Usutu virus (USUV) mainly circulate between the common house mosquito *Culex pipiens* (Linnaeus, 1758) as a primary vector species and several bird species as primary hosts [1]. Humans and other mammals like horses are dead-end hosts, but virus transmission from mosquitoes does occur. In 2018, the number of WNV infections spiked in 11 European Union (EU)/European Economic Area (EEA) member states, with the detection of 1605 human cases, including 166 lethal cases [2]. The detection of human infections with USUV is more sporadic, but recent studies found multiple native and invasive mosquitoes in Europe that were infected with USUV, increasing the potential to have many competent vectors in the future [3, 4]. Additionally, global warming will improve the conditions for arboviral replication and vector population growth [5, 6].

Insecticides against these vectors are used to locally reduce the likelihood of potential arboviral outbreaks. Vector species are controlled by distributing larvicides targeting the larval life stage and adulticides targeting the adult life stage of the mosquito. One of the most commonly used larvicides in Europe is *Bacillus thuringiensis israelensis* (Bti) [7]. Adult-targeting insecticides belong to the chemical classes pyrethroids, organochlorines, organophosphates and carbamates [8].

In the European Union (EU), biocides that can be used for mosquito control belong to the product type (PT) 18: “Insecticides, acaricides and products to control other arthropods” (Regulation No. 528/2012). For the successful approval of a biocide, two steps are required: firstly, active substance approval at the EU level, and secondly, product approval at the national level [9]. Active substances are divided into “new active substances” and “existing active substances”. “Existing active substances” are substances that were already on the market as active substances in biocidal products as of 14 May 2000. The EU regulation No. 1062/2014 lists all reported existing active substances until that date. Biocidal products that contain active substances that are not listed in the regulation may no longer be marketed. The “existing active substances” were evaluated in an EU programme with regard to risks to humans, animals and the environment. For those biocidal products only containing “existing active substances”, transitional arrangements are present, where the “existing active substances” need to be (dis)approved until 2024 [10]. When consulting the European Chemicals Agency (ECHA) database on 4 October 2022, 44 active substances were allowed as PT18 products by the European Union (Table 1; Additional file 4: Table S2) [11]. Additionally, chemical adulticides are rarely used for vector control in Europe, due to the risk of insecticide resistance in local vector populations [12]. There are five known mechanisms of insecticide resistance, namely target-site insensitivity, metabolic resistance, reduced penetration resistance, excretion and behavioural resistance, all reducing the effectiveness of insecticides. Epigenetic resistance has recently been discussed as the sixth insecticide resistance mechanism [13].

**Table 1:** Overview of the insecticides used during the WHO susceptibility tests with their approval as pesticides and insecticides according to the EU pesticides database and the ECHA database [18, 26]

	EU pesticides database	Approval end date	ECHA database	Approval end date
Carbamates				
Bendiocarb	X	–	✔PT18^a^	31 January 2024
Organochlorines				
DDT	X	–	X	–
Organophosphates				
Malathion	✔	30 April 2023	X	–
Pyrethroids				
Deltamethrin	✔	31 October 2023	✔PT18	30 September 2023
Permethrin	X	–	✔PT08^b^	30 April 2026
			✔PT18	30 April 2026
Other				
*Bacillus thuringiensis*	✔	30 April 2023	✔PT18	30 June 2026

^a^PT18 = product type insecticides, acaricides and products to control other arthropods

^b^PT08 = product type wood preservatives

The World Health Organization (WHO) developed a global plan to manage insecticide resistance, though the primary focus is on vector control against anopheline mosquitoes to combat malaria. The global plan for insecticide resistance management in malaria vectors is based on five pillars: (1) to plan and implement insecticide resistance management strategies in malaria-endemic countries, (2) to ensure proper, timely entomological and resistance monitoring with effective data management, (3) to develop new and innovative vector control tools, (4) to fill gaps in knowledge on mechanisms of insecticide resistance and the impact of current insecticide resistance management strategies, and (5) to ensure that enabling mechanisms (advocacy, human and financial resources) are in place [14]. It would be relevant to broaden this plan and integrate arboviral vector species.

In Belgium, a country in Central Europe, *Cx. pipiens* is extremely common and widespread, with the species being present at 698 of the 1000 monitored sites [15]. At the moment, Belgian *Cx. pipiens* are not controlled, but emerging case numbers of WNV and USUV in neighbouring countries may enforce vector control in the near future. Insecticide resistance in Belgian *Cx. pipiens* could have already arisen from the larvicide Bti, used against invasive *Aedes* vectors since 2014 [16]. Accidental exposure of Belgian *Cx. pipiens* to pesticides that are used for plant protection is also very likely (EU Regulation No. 1107/2009). Pesticides used in agriculture in Belgium with a known effect on mosquitoes are malathion, thiophanate-methyl and lambda-cyhalothrin [17–20]. In 2019, permethrin accounted for 0.5% and *Bacillus thuringiensis* for 4.4% of the total amount of pesticides used by public administrations. Those percentages translate into 13.5 kg of active substance for permethrin and 163.6 kg for *Bacillus thuringiensis*, respectively [21]. The Belgian *Cx. pipiens* might have been exposed to those or other agricultural pesticides during a sugar meal from treated plants or pesticides used in households and gardening. In addition, spraying of plants can cause direct exposure of adults or indirect exposure of larvae/pupae due to contamination of their breeding habitats. Because of these multiple scenarios for insecticide exposure of Belgian *Cx. pipiens* in their natural environment, we hypothesise that Belgian *Cx. pipiens* developed insecticide resistance against several insecticides.

The aim of this study was to assess the current status of insecticide resistance of *Cx. pipiens* as available from the literature. We further investigate whether the susceptibility status of Belgian *Cx. pipiens* mosquitoes is comparable to other *Cx. pipiens* populations because accidental environmental exposure to insecticides is a common global phenomenon [22, 23]. A detailed understanding of insecticide resistance in *Cx. pipiens* will support the rapid and sustainable development of integrative vector control strategies during arboviral outbreaks that involve *Cx. pipiens* as a vector.

## Methods

### Systematic literature review

The insecticide resistance status of the tested Belgian *Cx. pipiens* was compared to the insecticide resistance status of other *Cx. pipiens* populations worldwide by a systematic literature review. The PubMed database was used to find publications regarding the insecticide resistance status of *Cx. pipiens* mosquitoes, written in English and published up to 8 August 2022. The following search terms were all individually searched, following the Preferred Reporting Items for Systematic Reviews and Meta-Analyses (PRISMA) flow chart (Additional file 1: Fig S1): “Insecticide resistance *Culex pipiens”*, “WHO susceptibility test *Culex pipiens”*, “insecticide resistance bioassay *Culex pipiens”*, “Phenotypic resistance *Culex pipiens”*, “Malathion resistance *Culex pipiens”*, “Bendiocarb resistance *Culex pipiens”*, “DDT resistance *Culex pipiens”*, “Permethrin resistance *Culex pipiens”* and “Deltamethrin resistance *Culex pipiens”*. A total of 749 hits were found for the search terms. Based on the titles and key words, 263 publications were selected to read the abstract, and based on the abstract, 68 publications were read completely. Research papers were included in further bibliometric analysis if the following criteria were fulfilled: (1) use of WHO susceptibility tests with malathion, bendiocarb, DDT, permethrin and deltamethrin; and (2) use of *Cx. pipiens* mosquitoes in the WHO tests to detect insecticide resistance. Publications that obtained results with methods other than WHO tests to detect insecticide resistance and publications that reported insecticide resistance for *Culex* mosquitoes other than *Cx. pipiens* were excluded (Additional file 1: Fig. S1*).

This procedure resulted in 17 publications that fulfilled all criteria. An additional search on Google Scholar, following the same search method, yielded two additional publications that were used in this research.

### Mosquito collection and rearing

The *Cx. pipiens molestus* strain (20CPip.BE-ITMf.6) used for the experiments originated from larval collections in Hove (Belgium) in 2020. Larvae were collected from a rain barrel in a private garden, where no insecticides have been used since 2011, and the closest agricultural activity is a cornfield 500 m away. The bioform *molestus* was confirmed via multiplex polymerase chain reaction (PCR) with three primers: CQ11F2, MOLCQ11R and PIPCQ11R, PCR for *Cx. pipiens pipiens* with CQ11F2 and PIPCQ11R and PCR for *Cx. pipiens molestus* with CQ11F2 and MOLCQ11R. The Belgian *Cx. pipiens* were reared as a colony with overlapping generations in climatic cupboards (CPS-P530 Climatic Cabinet, RUMED Germany) at 27 °C with relative humidity of 80% and a 16:8 light/dark cycle at the Merian insectary of the Institute of Tropical Medicine (ITM), Antwerp, Belgium.

Prior to the experiments, egg rafts were collected from the colony, placed on humid cotton wool in Eppendorf tubes and stored at 8 °C for less than a week (until sufficient eggs were collected). Approximately 20 rafts were added to each of four plastic trays containing two litres of soft water. First-instar larvae were fed ad libitum on sieved TetraMin (Tetra, Germany) [24]. Second- to fourth-instar larvae were fed daily on Koi ministicks ad libitum. Pupae were collected in plastic cups filled with 80 ml of soft water, which were placed into 17 × 17 cm cages containing a sugar feeder. Adult density varied, with a maximum of 120 adults per cage.

### WHO susceptibility tests with five adulticides

A WHO test kit and insecticide-impregnated papers from Universiti Sains [University of Science] Malaysia (Malaysia) were used to conduct bioassays, as outlined by the WHO Guidelines for test procedures for insecticide resistance monitoring in malaria vector mosquitoes [25]. Following insecticides from the Universiti Sains Malaysia were used: bendiocarb 0.1%, dichlorodiphenyltrichloroethane (DDT) 4.0%, malathion 5.0%, deltamethrin 0.05% and permethrin 0.75%. Three out of five tested insecticides are currently approved in the European Union: bendiocarb, deltamethrin and permethrin. DDT and malathion are non-approved insecticides in the European Union (Table 1).

Twenty females between 2 and 5 days old were placed in every six resting tubes containing regular white paper. Six exposure tubes, from which two control tubes containing oil-treated paper and four tubes containing insecticide-impregnated paper, were attached to the resting tubes. All mosquitoes were transferred to the exposure tubes and placed in an upright position for 1 h at 27 °C. Afterwards, mosquitoes were transferred back to the resting tubes for 23 h with access to soaked cotton wool containing a 10% glucose dilution.

All mosquitoes were preserved after the WHO susceptibility test at −20 °C for a protein assay and glutathione S-transferase (GST) assay (for method and results see Additional file 2).

### WHO larvicidal susceptibility test with Bti

A Bti larvicidal bioassay was conducted as outlined in the WHO Guidelines for laboratory and field testing of mosquito larvicides [27]. A portion of Bti tablet (Neudorff, Germany) was crushed and dissolved in lukewarm softened water and mixed for 10–15 min. The concentrations of 0 mg L^−1^ (control), 0.02 mg L^−1^, 0.05 mg L^−1^, 0.10 mg L^−1^, 0.15 mg L^−1^, 0.20 mg L^−1^ and 0.30 mg L^−1^ were tested for larval toxicity in fourfold replication at 27 °C. Twenty late third- to early fourth-instar larvae were added to 100 ml of softened water for each concentration. After 24 h, larvae were checked and moribund and non-moving larvae were considered to be dead. Surviving larvae were stored at −20 °C for a protein assay and GST assay (for methods and results see Additional file 2).

### Data analysis

For each insecticide and control group during the WHO larvicidal susceptibility test with Bti and the WHO susceptibility tests with five adulticides, the mortality (%) was calculated by dividing the total number of dead mosquitoes by the total sample size. If the mortality of the control group ranged between 5 and 20%, a correction was made using Abbot’s formula.

$$\text{Observed mortality = }\frac{\text{Total number of dead mosquitoes}}{\text{Total sample size}}*100$$

$${\text{Corrected}} \, {\text{mortality}} = \frac{\% \, observed \, mortality-\% \, control \, mortality}{100-\% \, control \, mortality}*100$$

### WHO susceptibility tests with five chemical adulticides

Mortality was analysed according to the WHO protocol [25]: A mortality of 100–98% indicates susceptibility, 97.9–90% indicates possible resistance and < 90% mortality indicates resistance.

A database per insecticide was created using the mortality per country obtained from the systematic literature review. Literature data on insecticide resistance for *Cx. pipiens* and additional experimental insecticide resistance data for the Belgian population were plotted using Prism^®^ (version 9.3.1, GraphPad Software Inc., USA). A map used to visualise the results of the systematic literature review was created with Datawrapper [28].

### WHO larvicidal bioassay with Bti

Mortality was plotted against x-log-transformed Bti concentrations, and a lethal concentration 50% (LC_50_) and lethal concentration 95% (LC_95_) were calculated. The data were log-transformed and non–linear regression was performed using Prism^®^ (version 9.3.1, GraphPad Software Inc., USA).

## Results

### Insecticide resistance status of adult *Cx. pipiens*

**Fig. 1:**
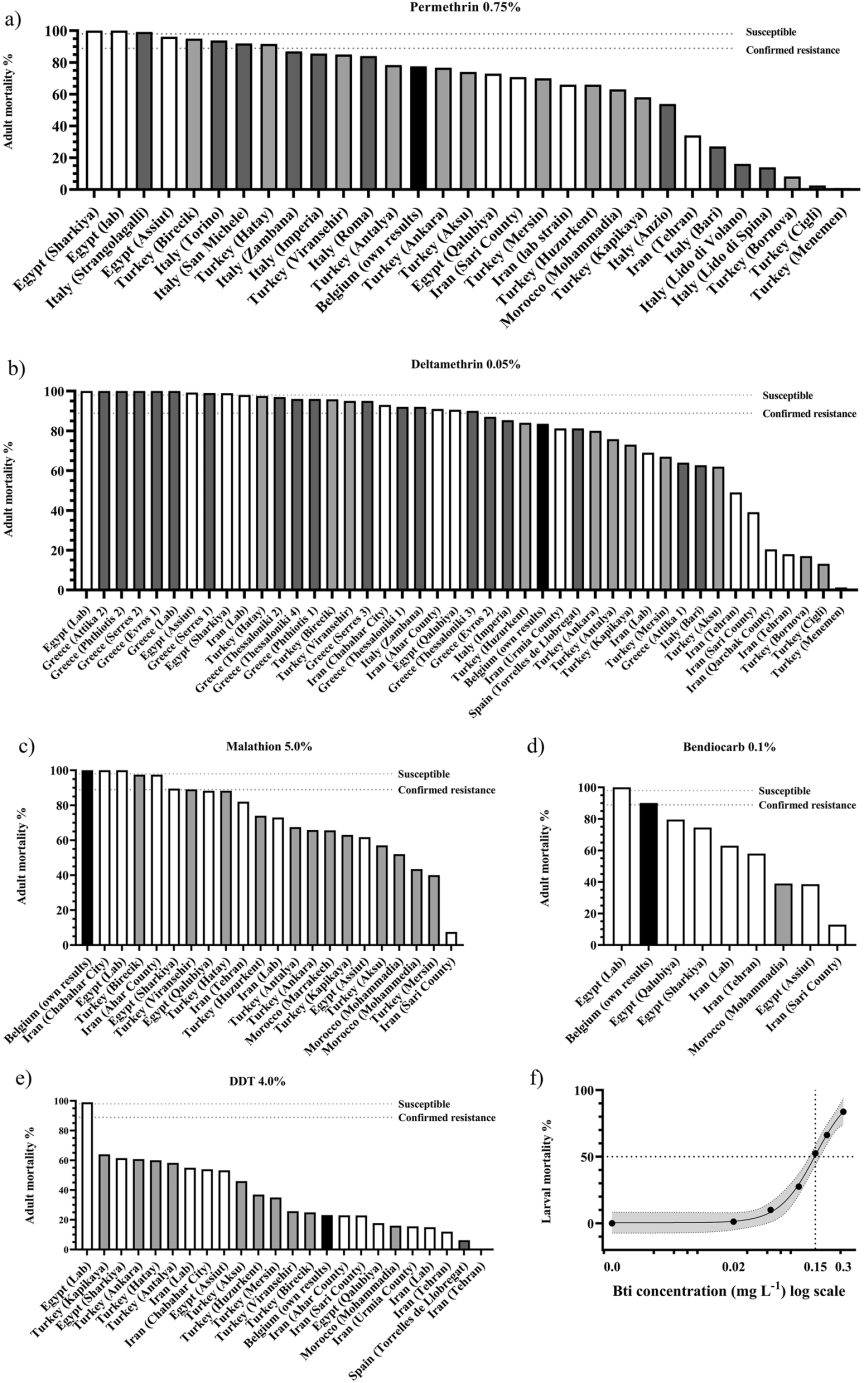
Insecticide resistance status in *Cx. pipiens*. Resistance towards **a** permethrin, **b** deltamethrin, **c** malathion, **d** bendiocarb, **e** DDT, **f** Bti. **f** WHO larvicidal susceptibility test with Bti on the Belgian population. Insecticides approved by the EU are **a**, **b**, **d**, **f**; insecticides for which the Belgian population is susceptible/possibly resistant are **c**, **d**; insecticides for which the Belgian population is resistant are **a**, **b**, **e**, **f**. Black indicates Belgian population, dark grey EU, light grey neighbouring countries to EU, white outside EU

**Fig. 2:**
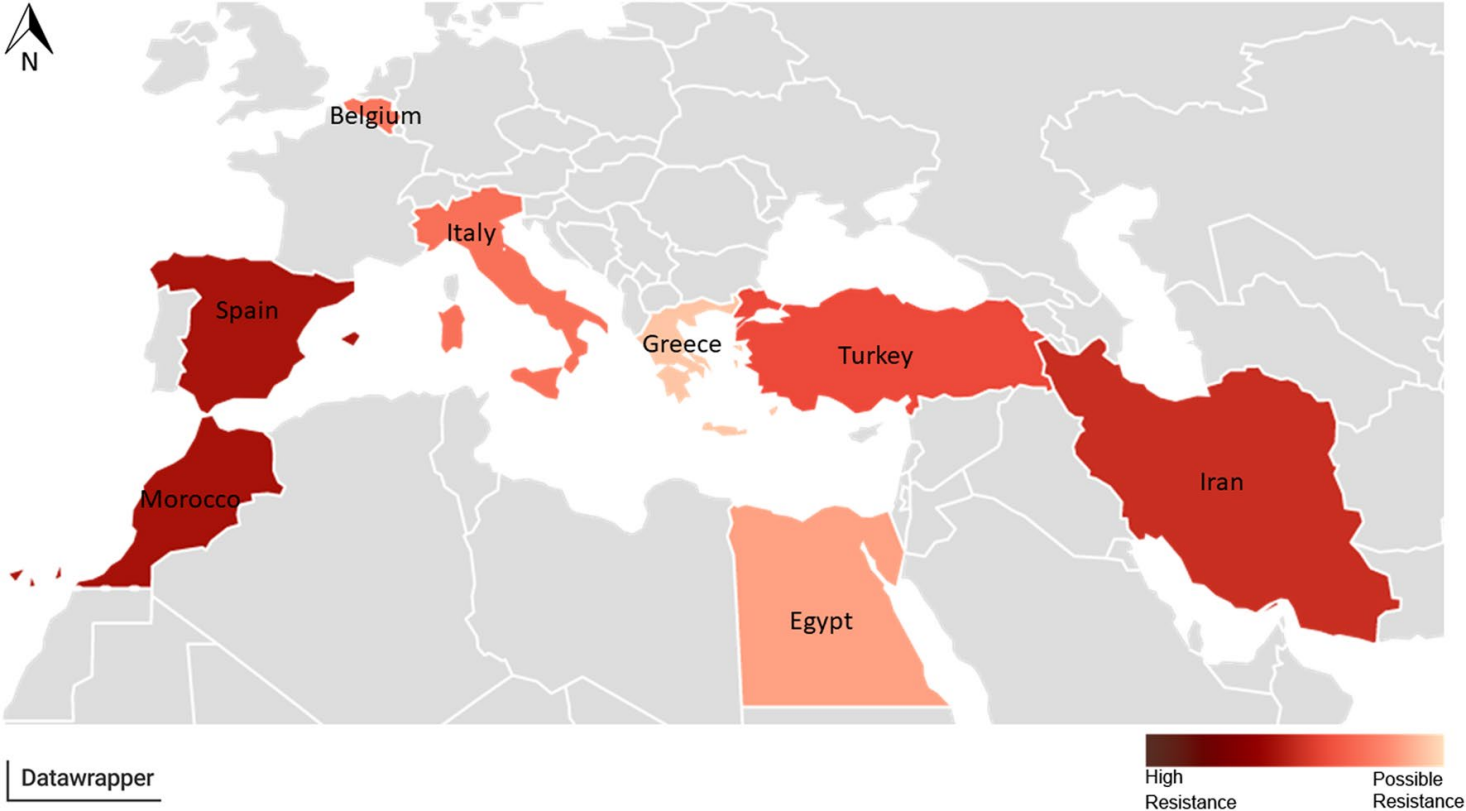
Countries coloured by insecticides resistance status of *Culex pipiens* for the five tested insecticides. Light red indicates possible resistance towards tested insecticides, dark red indicates high resistance towards tested insecticides

The systematic literature search combined with experimental data for a Belgian *Cx. pipiens* population revealed a broad geographic distribution of insecticide resistance in *Cx. pipiens* (Figs. 1, 2; Additional file 3: Table S1).

### Bendiocarb

Only a lab strain from Egypt was susceptible for bendiocarb, with mortality of 100% [29]. Other colonies from Egypt, Morocco and Iran have shown resistance, with mean mortality below 90% [30–33]. In comparison, the Belgian population showed possible resistance, with mean mortality of 90.1%.

### Permethrin

Only two populations, both from Egypt, indicated full susceptibility for permethrin, with mortality of 100% [29]. Possible resistance was found for six populations: one from Egypt (Assiut), two from Turkey (Birecik and Hatay) and three from Italy (Strangolagalli, Torino and San Michele) [34, 35]. Twenty-two other populations from Italy, Turkey, Egypt, Iran and Morocco indicated resistance towards permethrin [30–33, 36, 37]. In comparison, the Belgian population results in a mortality of 77.5%, indicating resistance.

### Deltamethrin

Complete susceptibility towards deltamethrin was observed in Egypt and Greece in six out of 44 populations tested [29, 38]. For 16 out of 44 populations, possible susceptibility was found, including populations from Egypt, Greece, Iran, Turkey and Italy [31–33, 35, 39–43]. Twenty-one populations from Greece, Italy, Iran, Spain and Turkey indicated resistance [34, 36, 37, 44]. In comparison, the Belgian population showed clear resistance towards deltamethrin with mean mortality of 83.5%.

### DDT

Only one population, a lab strain from Egypt, showed possible resistance for DDT. All other populations indicated resistance, including the Belgian population with 23.2% mean mortality for DDT [29–34, 37, 39–41, 44]

### Malathion

WHO susceptibility tests with malathion resulted in three fully susceptible populations: the Belgian population, a population from Iran (Chabahar City) and a lab strain from Egypt [29, 43]. Two populations, one from Turkey (Birecik) and one from Iran (Ahar County) showed possible resistance [34, 39]. Seventeen other populations from Egypt, Turkey, Morocco and Iran indicate resistance towards malathion [30, 32, 33, 37, 45].

### WHO susceptibility test with five adulticides

The Belgian *Cx. pipiens* developed insecticide resistance against at least three out of five insecticides (Table 2). The experimental population was resistant against deltamethrin, permethrin and DDT, as indicated by 83.5%, 77.5% and 23.2% mean mortality, respectively, with possible resistance against bendiocarb as indicated by mortality of 90.1%. The Belgian *Cx. pipiens* population was susceptible for malathion, as indicated by mortality of 100%.

**Table 2:** Insecticide susceptibility of Belgian *Cx. pipiens* as assessed with WHO susceptibility tests with five chemical insecticides and WHO larvicidal bioassay with Bti

Insecticide class	Insecticide	Discriminating concentration (%) 1-h exposure	Number of mosquitoes (n)	Mortality (%)	Status	Control	Number of mosquitoes (n)	Mortality (%)
Pyrethroid	Permethrin	0.75	80	77.5	R^c^	Silicone oil	40	0
	Deltamethrin	0.05	79	83.5	R	Silicone oil	40	2.5
Organophosphate	Malathion	5	84	100	S^a^	Olive oil	42	7.1
Carbamate	Bendiocarb	0.1	81	90.1	PR^b^	Olive oil	40	0
Organochlorin	DDT	4	82	23.2	R	Risella oil	42	0

Insecticide class	Insecticide	Number of mosquitoes (n)	LC50 (95% CI)	LC95 (95% CI)	Status	Control	Number of mosquitoes (n)	Mortality (%)
Biological	Bti	400	0.15 (0.11–1.00)	0.56 (0.25–18.16)	PR	Soft water	80	0

^a^*S* susceptible

^b^*PR* possible resistance

^c^*R* resistant

### WHO larvicidal susceptibility test with Bti

No mortality was observed in the control group of Belgian *Cx. pipiens*; hence, Abbot’s formula was not used. The LC_50_ of Bti for Belgian *Cx. pipiens* is 0.15 mg L^−1^ and LC_95_ 0.56 mg L^−1^, respectively (Fig. 1).

## Discussion

Our hypothesis on the universal distribution of insecticide resistance in *Cx. pipiens* mosquitoes could be confirmed by means of a combined experimental–bibliometric approach. To our knowledge, this is the first systematic literature review on phenotypic insecticide resistance in *Cx. pipiens* mosquitoes, while previous studies focused on single insecticide classes and different insecticide detection methods [46].

Bti, deltamethrin and permethrin are allowed to be used as insecticides against mosquitoes in the EU with an approval until 2026, 2023 and 2026, respectively (Table 1). Unfortunately, the Belgian *Cx. pipiens* mosquitoes revealed resistance against the EU-approved insecticides permethrin, deltamethrin and the banned DDT. In addition, Bti provoked low larvicidal activity in the tested Belgian *Cx. pipiens* population.

### Phenotypic insecticide resistance of *Culex* species

The Belgian *Cx. pipiens* developed insecticide resistance against at least three out of five insecticides (Table 2). Insecticide resistance in *Cx. pipiens* has also been detected with WHO susceptibility tests in Italy, Spain, Greece, Egypt, Morocco, Turkey and Iran [29–45]. In Spain, the WHO susceptibility test (in compliance with the CDC bottle test) revealed the insecticide resistance of *Cx. pipiens* populations to deltamethrin, permethrin, bendiocarb, lambda-cyhalothrin, propoxur, pirimiphos-methyl and DDT [44]. Also *Cx. pipiens pallens* (four out of five Chinese populations) revealed resistance to deltamethrin, with the exception of a susceptible lab strain [47].

The sister species *Cx. quinquefasciatus* from Pemba Island, Zanzibar, is resistant to all insecticides (DDT, permethrin, deltamethrin, lambda-cyhalothrin and bendiocarb) tested with the WHO susceptibility test [48]. In line, four *Cx. quinquefasciatus* populations from Cameroon showed insecticide resistance against DDT, permethrin, deltamethrin, bendiocarb and malathion (with mortalities below 15.0%). Additionally, three out of four populations were resistant against malathion [49]. In Zambia, *Cx. quinquefasciatus* mosquitoes were used in CDC bottle tests, another bioassay to detect phenotypic resistance. The *Cx. quinquefasciatus* field mosquitoes that were tested had a lower knockdown rate for DDT than the susceptible mosquitoes. Even after exposure to a higher concentration of DDT, the knockdown rate was lower than expected, indicating the severity of the insecticide resistance.

Bti exposure (Neudorff, ground-up Bti tablet) of the Belgian *Cx. pipiens* population showed a very high LC_50_ result compared to the literature. The LC_50_ value for the Belgian *Cx. pipiens* strain, 0.15 mg L^−1^, is five times higher than the LC_50_ value for a field collection from Chico, California (VectoBac WDG, water-dispersible granules) and 10 times higher than the LC_50_ value found for a strain from Syracuse, New York (self-made powder from Bti IPS-80 culture) [50, 51]. When comparing the Belgian LC_50_ result with results from Cyprus, there is even a 50-fold difference with the highest LC_50_ result from Cyprus (VectoBac SL, aqueous suspension) [52]. These comparisons are remarkable, although it is important to note that variation in formulation and toxicity of the different Bti products can partly explain these differences in LC_50_ results. In addition to the low larval susceptibility to the biocide Bti, larval resistance against chemical insecticides has been confirmed in an Italian field population, where the RRLC_50_ (Resistance Ratio Lethal Concentration 50%) for the larvicide diflubenzuron increased from 32- to 128-fold over the span of 1 year [53]. This diflubenzuron resistance in Italian *Cx. pipiens* was confirmed by diflubenzuron-associated mutations I1043L and I1043M in *Culex* populations [54].

### Operational aspects

According to our results for the Belgian *Cx. pipiens* population, malathion would be the only insecticide that should be used in case of an arboviral outbreak that involves *Culex* mosquitoes (e.g., WNV) in Belgium. However, malathion belongs to the “existing active substances” since it was not on the market before the 14th of May 2000. A complete dossier was not submitted in order to include malathion in the “existing active substances” list. Accordingly, malathion is not available on the EU market anymore as a biocidal product (Table 1). However, malathion was used in France (French Guiana) against adult mosquitoes after approval by the EU in 2009. This was possible because in Directive 98/8/EC of the European Parliament (Regulation before No 528/2012) article 15 (1) states that it is allowed to place biocidal products temporarily (120 days) on the market after the approval of the commission because of unforeseen danger. The new EU regulation No 528/2012 even would allow such a derogation for 180 days (Article 55). This means that in case of a WNV outbreak, the European Union or Belgium may allow the use of malathion for 180 days due to the danger to public and animal health. We recommend that malathion should be evaluated to be approved in general under the EU Regulation No 528/2012.

Next to malathion, bendiocarb achieved the highest mortality rates on *Cx. pipiens* (90.1%) in our study. As the approval of bendiocarb will end soon (on 31 January 2024), we suggest an extension of the approval to make it possible to use bendiocarb for vector control in the future. If the approval is not extended, bendiocarb could potentially be used with an exceptional approval for 180 days as described for malathion (EU regulation: 528/2012 Article 55).

Notably, all the approvals of insecticides, as stated by the EU pesticides database and ECHA database, will expire within the next 8 years. It is of great importance to improve the knowledge regarding the insecticide resistance of native and invasive mosquitoes in Europe in order to guide the (dis)approval of certain insecticides if needed during an arboviral outbreak. The importance of good policy-making regarding insecticide resistance is clearly illustrated by the example of the banned insecticide DDT. The insecticide DDT (half-life = 6200 days, according to the pesticide properties database of the University of Hertfordshire) has been banned since the seventies, but *Cx. pipiens* mosquitoes from various geographic origins around the globe show strong resistance against DDT to this day [55].

### Limitations of the study

Our study provides the first overview of the phenotypic insecticide resistance status of *Cx. pipiens* populations worldwide and in particular from Belgium. However, more WHO susceptibility tests with different *Culex* populations from across Belgium and other European countries are required to form a complete picture of the insecticide resistance status of *Cx. pipiens*. Genetic resistance assays are also used to monitor the insecticide resistance status of mosquitoes, however, not all genetic markers that are involved in insecticide resistance are known yet and the genetic markers that are known are not always fully able to explain the phenotypic resistance [56]. Literature reports indicate that the resistance status of *Cx. pipiens* is associated with genetic mutations typically associated with insecticide resistance. In *Cx. pipiens* from Greece, both a relatively high frequency of the knockdown resistance (*kdr*) L1014F resistance mutation and resistance to deltamethrin have been detected [38]. Likewise, in different *Cx. quinquefasciatus* populations from Guadeloupe, insecticide resistance against deltamethrin was observed along with the detection of a high frequency of the L1014F *kdr* mutation [57]. Regarding the Belgian population, our findings are supported by a study that confirmed the presence of *kdr* L1014F and *ace*-1 G119S resistance mutations in *Cx. pipiens* samples collected from four different locations in Belgium [58]. Resistance mechanisms could not be elucidated in this study. An initial experiment does not indicate elevated levels of GST-specific activity after exposure to insecticides. Further testing is needed to find the resistance mechanism behind the observed phenotypic resistance.

## Conclusion

This research provides the first evidence of phenotypic insecticide resistance in Belgian *Culex pipiens*. As it is clear that insecticide resistance in *Culex* populations is a global phenomenon, we propose the implementation of a public repository of the insecticide resistance status in *Culex* populations which would allow vector control managers easier access and overview of insecticide resistance data for *Culex* mosquitoes, as is already in place for insecticide resistance in anopheline and aedine mosquitoes on websites such as www.irmapper.com. This information can then be used to inform (dis)approval of insecticides to prevent a further increase in insecticide resistance against certain insecticides in the EU and abroad. The translation of these alarming results on limited efficiency and availability of insecticides into EU legislation and the development of new non-chemical vector control tools are the next logical steps to counter the widespread insecticide resistance in *Culex* populations.

## Supplementary Information

**Additional file 1: Fig. S1.** PRISMA flow chart.

**Additional file 2: Fig. S2.** Material and method, results and conclusion of the protein and GST assays.

**Additional file 3: Table S1.** Insecticide resistance tested via WHO susceptibility tests in Belgium, Egypt, Greece, Iran, Italy, Morocco, Spain and Turkey.

**Additional file 4: Table S2.** Overview of all active substances that are approved as PT18 products according to the ECHA database on 4/10/2022.

## Data Availability

Data supporting the conclusions of this article are included within the article.

## Abbreviations

Bti: *Bacillus thuringiensis israelensis*
*Cx.*: *Culex*
DDT: Dichlorodiphenyltrichloroethane
EU/EEA: European Union/European Economic Area
GST: Glutathione *S*-transferase
ITM: Institute of Tropical Medicine
kdr: Knockdown resistance
LC50: Lethal concentration that kills 50% of the larvae
RR: Resistance ratio
WHO: World Health Organization
