# Supplementary material for: Phenotypic insecticide resistance status of the Culex pipiens complex: a European perspective
Source: Parasit Vectors. 2022 Nov 12;15:423. doi: 10.1186/s13071-022-05542-x (PMC9652947; doi:10.1186/s13071-022-05542-x)
Supplement: Supplementary file 1 — Additional file 1: Fig. S1. PRISMA flow chart. [file 13071_2022_5542_MOESM1_ESM.docx]

**Additional file 1**

Studies included in review

(n = 17 )

**Identification of studies via PubMed**

**Screening**

Records screened

(n = 749)

Records excluded based on title and key words

(n = 486 )

Abstracts screened

(n = 263 )

Records excluded based on pre-set requirements (*)

(n = 195 )

Reading of the full article

(n = 68 )

Removal of duplicates and publications that did not meet the requirement and/or did not state clear results
(n = 51)

**Included**

**Identification**

Records identified from:

Databases (n = 749)

Fig. S1 Prisma flow chart
