# Supplementary material for: Phenotypic insecticide resistance status of the Culex pipiens complex: a European perspective
Source: Parasit Vectors. 2022 Nov 12;15:423. doi: 10.1186/s13071-022-05542-x (PMC9652947; doi:10.1186/s13071-022-05542-x)
Supplement: Supplementary file 2 — Additional file 2: Fig. S2. Material and method, results and conclusion of the protein and GST assays. [file 13071_2022_5542_MOESM2_ESM.docx]

**Additional file 2**

This additional file contains the material and method, results and conclusion of the protein and GST assays performed on *Cx. pipiens* samples from the WHO susceptibility tests. Control mosquitoes, not exposed to any insecticides, and insecticide exposed mosquitoes were used to measure the protein concentration and GST specific activity of each sample to check if elevated levels of GST specific activity, related to the metabolic insecticide resistance mechanism, would be involved in the observed phenotypic insecticide resistance of Belgian *Cx. pipiens*.

**Material and method**

Only mosquitoes used during the WHO susceptibility test were tested for the biochemical assays. Protein assays were conducted to correct the GST specific activity. GST assay was performed to determine GST specific activity to account for elevated levels of detoxification. In total ten surviving controls and eighteen surviving exposed mosquitoes, from which six from the test with deltamethrin, six from permethrin and six from DDT. Mosquitoes were stored at -20°C and placed in individual Eppendorf tubes. Two glass beads and 300µl of sodium sulphate 2% w/v were added per Eppendorf tube. The tubes were homogenized for five seconds at 4.5 speed and centrifuged briefly. All samples were stored at -80°C.

The protein assay was modified from the Bradford protein assay. First a standard curve was obtained in duplicate with 0, 1, 5, 10, 15 and 20µg BSA and complemented with distilled water to a total of 800µl per tube. Homogenized samples were taken out of the -80°C freezer, 50µl per sample was transferred to a new tube and 750µl distilled water was added. To both standard curve and experiment samples, 200µl of Rotiquant (Carl Roth GmbH, Germany) dye reagent was added. All samples were vortexed and briefly centrifuged before transferring 200µl per sample to a 96-well-plate. Absorbance was read at 595nm within one hour via SkanIt Software RE for Microplate Readers.

**Results**

No significant difference between the control group and insecticide exposed mosquitoes was found in GST specific activity (Fig. S1)

Fig. S2 GST specific activity for control group, deltamethrin, permethrin and DDT in µmol/min/mg protein.

**Conclusion**

This initial experiment does not indicate elevated levels of GST specific activity after exposure to insecticides. Further testing is needed to find the resistance mechanism behind the observed phenotypic resistance.
