# Supplementary material for: Phenotypic insecticide resistance status of the Culex pipiens complex: a European perspective
Source: Parasit Vectors. 2022 Nov 12;15:423. doi: 10.1186/s13071-022-05542-x (PMC9652947; doi:10.1186/s13071-022-05542-x)
Supplement: Supplementary file 3 — Additional file 3: Table S1. Insecticide resistance tested via WHO susceptibility tests in Belgium, Egypt, Greece, Iran, Italy, Morocco, Spain and Turkey. [file 13071_2022_5542_MOESM3_ESM.docx]

**Additional file 3**

**Table S1 Insecticide resistance tested via WHO susceptibility tests in Belgium, Egypt, Greece, Iran, Italy, Morocco, Spain and Turkey.**

|  | Permethrin 0.75% | Deltametrhin 0.05% | Malathion 5% | Bendiocarb 0.1% | DDT 4% |
| --- | --- | --- | --- | --- | --- |
| Belgium (own results) | 77.5 | 83.54 | 100 | 90.12 | 23.17 |
| Egypt (Assiut) | 96.1 | 99.2 | 61.7 | 38.6 | 53.3 |
| Egypt (lab) | 100 | 100 | 100 | 100 | 99 |
| Egypt (Qalubiya) | 72.9 | 90.6 | 88.3 | 79.6 | 17.8 |
| Egypt (Sharkiya) | 100 | 98.9 | 89.5 | 74.5 | 61.5 |
| Greece (Attika 1) | NA | 64 | NA | NA | NA |
| Greece (Attika 2) | NA | 100 | NA | NA | NA |
| Greece (Evros 1) | NA | 100 | NA | NA | NA |
| Greece (Evros 2) | NA | 87 | NA | NA | NA |
| Greece (Lab) | NA | 100 | NA | NA | NA |
| Greece (Phthiotis 1) | NA | 96 | NA | NA | NA |
| Greece (Phthiotis 2) | NA | 100 | NA | NA | NA |
| Greece (Serres 1) | NA | 99 | NA | NA | NA |
| Greece (Serres 2) | NA | 100 | NA | NA | NA |
| Greece (Serres 3) | NA | 95 | NA | NA | NA |
| Greece (Thessaloniki 1) | NA | 92 | NA | NA | NA |
| Greece (Thessaloniki 2) | NA | 97 | NA | NA | NA |
| Greece (Thessaloniki 3) | NA | 90 | NA | NA | NA |
| Greece (Thessaloniki 4) | NA | 96 | NA | NA | NA |
| Iran (Ahar County) | NA | 91 | 97.5 | NA | 23 |
| Iran (Chabahar City) | NA | 93 | 100 | NA | 54 |
| Iran (lab strain) | 66 | 69 | 73 | 63 | 15 |
| Iran (Qarchak County) | NA | 20.4 | NA | NA | NA |
| Iran (Sari County) | 70.73 | 39.08 | 7.52 | 12.94 | 22.98 |
| Iran (Tehran) | 34 | 18 | 82 | 58 | 12 |
| Iran (Urmia County) | NA | 81.21 | NA | NA | 15.62 |
| Italy (Anzio) | 53.85 | NA | NA | NA | NA |
| Italy (Bari) | 27.1 | 62.71 | NA | NA | NA |
| Italy (Imperia) | 85.57 | 85.34 | NA | NA | NA |
| Italy (Lido di Spina) | 14 | NA | NA | NA | NA |
| Italy (Lido di Volano) | 16.16 | NA | NA | NA | NA |
| Italy (Roma) | 84 | NA | NA | NA | NA |
| Italy (San Michele) | 91.95 | NA | NA | NA | NA |
| Italy (Strangolagalli) | 99.18 | NA | NA | NA | NA |
| Italy (Torino) | 93.75 | NA | NA | NA | NA |
| Italy (Zambana) | 86.92 | 92 | NA | NA | NA |
| Morocco (Marrakech) | NA | NA | 65.6 | NA | NA |
| Morocco (Mohammadia) | 63 | NA | 43.5 | 39 | 16 |
| Spain (Torrelles de Llobregat) | NA | 81.2 | NA | NA | 6.3 |
| Turkey (Aksu) | 74 | 62 | 57 | NA | 46 |
| Turkey (Ankara) | 76.6 | 80 | 65.8 | NA | 60.8 |
| Turkey (Antalya) | 78.3 | 75.8 | 67.5 | NA | 58.3 |
| Turkey (Birecik) | 95 | 95.8 | 97.5 | NA | 25 |
| Turkey (Bornova) | 8.15 | 16.98 | NA | NA | NA |
| Turkey (Cigli) | 2.52 | 13.12 | NA | NA | NA |
| Turkey (Hatay) | 91.6 | 97.5 | 88.3 | NA | 60 |
| Turkey (Huzurkent) | 66 | 84 | 74 | NA | 37 |
| Turkey (Kapikaya) | 58 | 73 | 63 | NA | 64 |
| Turkey (Menemen) | 0.96 | 1.24 | NA | NA | NA |
| Turkey (Mersin) | 70 | 67 | 40 | NA | 35 |
| Turkey (Viransehir) | 85 | 95 | 89.1 | NA | 25.8 |
