# Supplementary material for: Phenotypic insecticide resistance status of the Culex pipiens complex: a European perspective
Source: Parasit Vectors. 2022 Nov 12;15:423. doi: 10.1186/s13071-022-05542-x (PMC9652947; doi:10.1186/s13071-022-05542-x)
Supplement: Supplementary file 4 — Additional file 4: Table S2. Overview of all active substances that are approved as PT18 products according to the ECHA database on 4/10/2022. [file 13071_2022_5542_MOESM4_ESM.docx]

**Additional file 4 Table S2 Overview of all active substances that are approved as PT18 products according to the ECHA database on 4/10/2022.**

| **Substance name** | **EC/list no** | **CAS no** | **Product type** | **Approval start date** | **Approval end date** | **Evaluating competent authority** | **Approval/assessment status** | **Related authorised biocidal products** |
| --- | --- | --- | --- | --- | --- | --- | --- | --- |
| Clothianidin | 433-460-1 | 210880-92-5 | PT18^a^ | 01/10/2016 | 30/09/2026 | Germany | Approved | 4 |
| Cypermethrin | -;  257-842-9 | 52315-07-8 | PT18 | 01/06/2020 | 31/05/2030 | Belgium | Approved - Other updates in progress | - |
| Cyphenothrin | -;  254-484-5 | 39515-40-7 | PT18 | 01/02/2020 | 31/01/2030 | Greece | Approved | - |
| Cyfluthrin | -;  269-855-7 | 68359-37-5 | PT18 | 01/03/2018 | 28/02/2028 | Germany | Approved | 11 |
| Hexaflumuron | -;  401-400-1 | 86479-06-3 | PT18 | 01/04/2017 | 30/09/2024 | Greece | Approved - Renewal in progress | - |
| 1R-trans phenothrin | -;  247-431-2 | 26046-85-5 | PT18 | 01/09/2015 | 31/08/2025 | Ireland | Approved | 31 |
| Piperonyl butoxide/PBO | -;  200-076-7 | 51-03-6 | PT18 | 01/07/2018 | 30/06/2028 | Greece | Approved | 16 |
| alpha-Cypermethrin | - | 67375-30-8 | PT18 | 01/07/2016 | 30/06/2026 | Belgium | Approved | 14 |
| Imiprothrin | -;  428-790-6 | 72963-72-5 | PT18 | 01/07/2019 | 30/06/2029 | United Kingdom | Approved | 1 |
| Abamectin | - | 71751-41-2 | PT18 | 01/07/2013 | 30/06/2023 | Netherlands | Approved | 1 |
| Acetamiprid | - | 135410-20-7 | PT18 | 01/02/2020 | 31/01/2027 | Belgium | Approved | - |
| Aluminium phosphide releasing phosphine | -;  244-088-0 | 20859-73-8 | PT18 | 01/02/2012 | 31/07/2024 | Germany | Approved - Renewal in progress | 3 |
| Bacillus sphaericus 2362, strain ABTS-1743 | - | 143447-72-7 | PT18 | 01/07/2016 | 30/06/2026 | Italy | Approved | 1 |
| Bacillus thuringiensis subsp. israelensis serotype H14, strain AM65-52 | - | - | PT18 | 01/10/2013 | 30/09/2023 | Italy | Approved - Renewal in progress | 11 |
| Bacillus thuringiensis subsp. israelensis, strain SA3A | - | - | PT18 | 01/07/2016 | 30/06/2026 | Italy | Approved | - |
| Bacillus thuringiensis subsp. kurstaki, strain ABTS-351 | - | - | PT18 | 01/03/2017 | 28/02/2027 | France | Approved | 1 |
| Bendiocarb | -;  245-216-8 | 22781-23-3 | PT18 | 01/02/2014 | 31/01/2024 | United Kingdom | Approved | 2 |
| Carbon dioxide | 204-696-9 | 124-38-9 | PT18 | 01/11/2012 | 31/10/2022 | France | Approved | 1 |
| Decanoic acid | -;  206-376-4 | 334-48-5 | PT18 | 01/09/2015 | 31/08/2025 | Austria | Approved | 1 |
| Deltamethrin | -;  258-256-6 | 52918-63-5 | PT18 | 01/10/2013 | 30/09/2023 | Sweden | Approved - Renewal in progress | 94 |
| Diflubenzuron | -;  252-529-3 | 35367-38-5 | PT18 | 01/02/2015 | 31/01/2025 | Sweden | Approved | 8 |
| Dinotefuran | - | 165252-70-0 | PT18 | 01/06/2015 | 30/11/2024 | Belgium | Approved - Renewal in progress | 2 |
| Epsilon-Momfluorothrin | - | 1065124-65-3 | PT18 | 01/07/2017 | 30/06/2027 | United Kingdom | Approved | - |
| Etofenprox | -;  407-980-2 | 80844-07-1 | PT18 | 01/07/2015 | 30/06/2025 | Austria | Approved | 7 |
| Fipronil | -;  424-610-5 | 120068-37-3 | PT18 | 01/10/2013 | 30/09/2023 | France | Approved | 7 |
| Hydrogen cyanide | -;  200-821-6 | 74-90-8 | PT18 | 01/10/2014 | 30/09/2024 | Czech Republic | Approved | 1 |
| Imidacloprid | -;  428-040-8 | 138261-41-3 | PT18 | 01/07/2013 | 30/06/2023 | Germany | Approved - Renewal in progress | 62 |
| Indoxacarb (enantiomeric reaction mass S:R 75:25) | - | - | PT18 | 01/01/2010 | 30/06/2024 | France | Approved - Renewal in progress | 3 |
| Kieselgur (diatomaceous earth) | 612-383-7 | 61790-53-2 | PT18 | 01/11/2018 | 31/10/2028 | France | Approved | - |
| Lambda-cyhalothrin | -;  415-130-7 | 91465-08-6 | PT18 | 01/10/2013 | 30/09/2023 | Greece | Approved - Renewal in progress | 5 |
| Magnesium phosphide releasing phosphine | -;  235-023-7 | 12057-74-8 | PT18 | 01/02/2012 | 31/07/2024 | Germany | Approved - Renewal in progress | 4 |
| Margosa extract from the kernels of Azadirachta Indica extracted with water and further processed with organic solvents | -;  283-644-7 | 84696-25-3 | PT18 | 01/05/2014 | 30/04/2024 | Germany | Approved | 2 |
| Metofluthrin | - | 240494-71-7 | PT18 | 01/05/2011 | 31/10/2023 | Ireland | Approved - Renewal in progress | 2 |
| Cyromazine | -;  266-257-8 | 66215-27-8 | PT18 | 01/01/2018 | 31/12/2027 | Greece | Approved | - |
| Octanoic acid | -;  204-677-5 | 124-07-2 | PT18 | 01/09/2015 | 31/08/2025 | Austria | Approved | 1 |
| Permethrin | -;  258-067-9 | 52645-53-1 | PT18 | 01/05/2016 | 30/04/2026 | Ireland | Approved | 39 |
| Pyriproxyfen | -;  429-800-1 | 95737-68-1 | PT18 | 01/02/2015 | 31/01/2025 | Netherlands | Approved | 9 |
| Pyrogenic, synthetic amorphous, nano, surface treated silicon dioxide | -;  272-697-1 | 68909-20-6 | PT18 | 01/11/2018 | 31/10/2028 | France | Approved | 1 |
| S-Methoprene | - | 65733-16-6 | PT18 | 01/09/2015 | 31/08/2025 | Ireland | Approved | 50 |
| Spinosad | -;  434-300-1 | 168316-95-8 | PT18 | 01/11/2012 | 31/10/2022 | Netherlands | Approved - Renewal in progress | 37 |
| Sulfuryl fluoride | -;  220-281-5 | 2699-79-8 | PT18 | 01/07/2011 | 31/12/2023 | Sweden | Approved - Renewal in progress | 2 |
| Synthetic amorphous silicon dioxide (nano) | -;  231-545-4 | 112926-00-8 | PT18 | 01/11/2015 | 31/10/2025 | France | Approved | 1 |
| Thiamethoxam | -;  428-650-4 | 153719-23-4 | PT18 | 01/02/2015 | 31/01/2025 | Spain | Approved | 2 |
| Transfluthrin | -;  405-060-5 | 118712-89-3 | PT18 | 01/11/2015 | 31/10/2025 | Netherlands | Approved | 56 |

^a^ = Product type 18: Insecticides, acaricides and products to control other arthropods
